# An enhanced real-time human pose estimation method based on modified YOLOv8 framework

**DOI:** 10.1038/s41598-024-58146-z

**Published:** 2024-04-05

**Authors:** Chengang Dong, Guodong Du

**Affiliations:** Nanjing University of Aeronautics and Astronautics, Nanjing, 210000 Jiangsu China

**Keywords:** Deep learning, Human pose estimation, Attention mechanisms, YOLOv8, Feature pyramid network, Computational science, Computer science, Software, Imaging and sensing

## Abstract

The objective of human pose estimation (HPE) derived from deep learning aims to accurately estimate and predict the human body posture in images or videos via the utilization of deep neural networks. However, the accuracy of real-time HPE tasks is still to be improved due to factors such as partial occlusion of body parts and limited receptive field of the model. To alleviate the accuracy loss caused by these issues, this paper proposes a real-time HPE model called ${\textbf {CCAM-Person}}$ based on the YOLOv8 framework. Specifically, we have improved the backbone and neck of the YOLOv8x-pose real-time HPE model to alleviate the feature loss and receptive field constraints. Secondly, we introduce the context coordinate attention module (CCAM) to augment the model’s focus on salient features, reduce background noise interference, alleviate key point regression failure caused by limb occlusion, and improve the accuracy of pose estimation. Our approach attains competitive results on multiple metrics of two open-source datasets, MS COCO 2017 and CrowdPose. Compared with the baseline model YOLOv8x-pose, CCAM-Person improves the average precision by 2.8% and 3.5% on the two datasets, respectively.

## Introduction

Real-time 2D Human Pose Estimation (HPE) constitutes a pivotal undertaking in the realm of computer vision, aiming to quickly infer the spatiotemporal arrangement of human keypoints, such as the head, shoulders, arms, and legs, from images or video frames and subsequently deduce their poses, such as bending, stretching, or rotating. Real-time 2D HPE plays a crucial role in various applications, including pose tracking, action recognition, virtual reality, and surveillance systems. By achieving accurate 2D HPE, we can obtain detailed information about human poses and actions, which can support computers in performing more complex human-computer interaction tasks.

HPE tasks mainly consist of two types: Single-person Pose Estimation (SPE) and Multi-person Pose Estimation (MPE). SPE focuses on mining the pose features of individual persons, and thus the model only needs to identify and regress the keypoints and skeleton information of the target person. These information typically include the category, relative positions, and confidences of the keypoints. In contrast, MPE involves detecting and estimating the poses of multiple individuals from an image. It aims to simultaneously locate and recognize the keypoints of multiple people and the posture connections between them. MPE tasks require addressing challenges such as occlusions, occluded body parts, and scale variations to obtain accurate and robust multi-person pose estimation results. MPE tasks have broader applications and deal with more complex scenarios, which are the main focus of this study.

**Figure 1:**
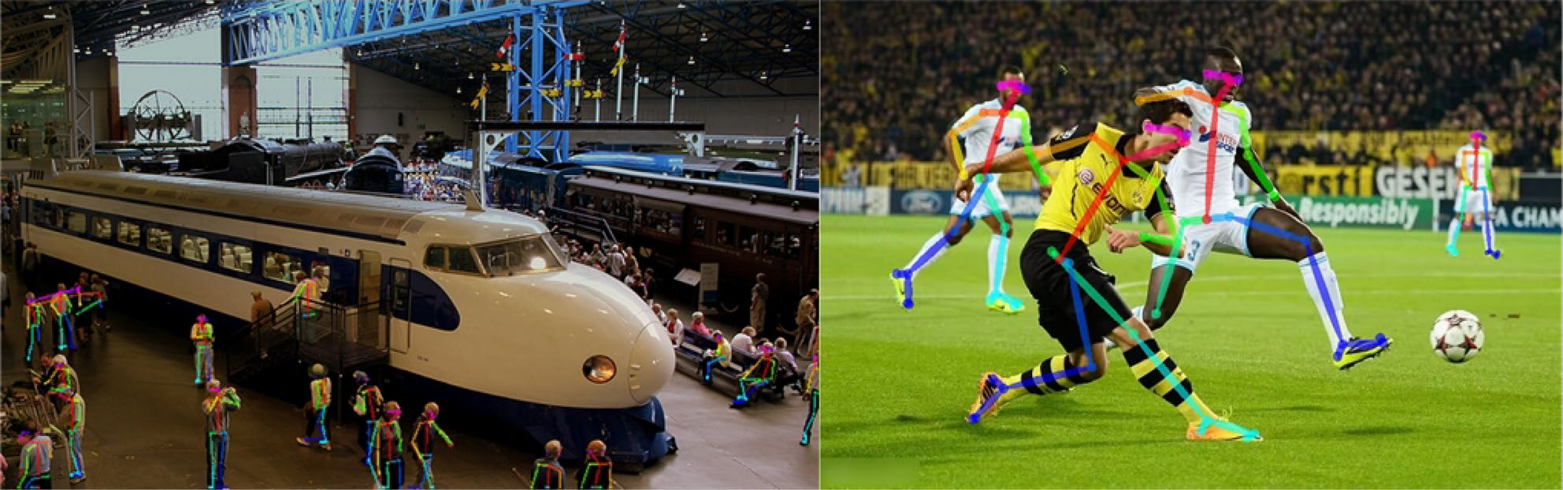
Current issues in multi-person pose estimation task. In the left image, attributable to the limited receptive field of the model, some keypoints of less prominent individuals in the image are not fully detected. In the right image, occlusions between individuals’ limbs lead to inaccurate regression of some keypoints, resulting in a decrease in pose estimation accuracy.

In recent years, a plethora of real-time 2D MPE models based on deep learning have emerged successively. These models^1–4^ employ deep neural networks as the basic architecture and further improve the regression capability of the models towards human keypoints through network structure modifications and post-processing optimizations^5,6^. To enhance the real-time pose estimation performance, researchers have adopted various strategies to reduce the inference cost of the network, such as lightweight network architectures^7,8^, weight sharing^9^, and spatial pyramid pooling^10^. Furthermore, certain approaches have endeavored to integrate MPE with other objectives, including but not limited to object detection and image segmentation, so as to cater to a broader spectrum of practical situations^11,12^. Despite the remarkable achievements made by deep learning-based real-time 2D HPE methods, some challenges and technical difficulties still exist, as shown in Fig. 1. First, a considerable multitude of stacked convolution or pooling modules in traditional network structures are prone to losing information from low-level features and limited receptive fields. Therefore, establishing more effective feature fusion mechanisms to improve the real-time keypoint regression capability of the model remains a challenging task. Second, the occurrence of entangled or occluded body parts can lead to the failure of regressing the corresponding keypoints. This phenomenon is also a current issue worthy of exploration.

The YOLO series techniques^11,13–15^ have served as popular models for visual comprehension and have assumed a significant role across diverse applications in real-time computer vision in recent years. Compared to its previous generations^14–16^, the latest YOLOv8^17^ demonstrates more powerful performance in terms of accuracy and speed and introduces the best-performing model, YOLOv8x-pose, specifically for the HPE task. The YOLOv8x-pose model utilizes the Path Aggregation Network (PANet)^18^ to construct a feature pyramid for comprehensive feature fusion across different receptive fields. Additionally, inspired by the Efficient Layer Aggregation Network (ELAN)^19^, YOLOv8x-pose further increases the receptive field of the backbone network at different levels. Furthermore, YOLOv8x-pose adopts the Task-Aligned Assigner^20^ proposed in YOLOX^21^ to replace the complex non-maximum suppression (NMS) process, thereby further improving the computational efficiency of the model’s inference.

However, due to the feature loss and receptive field limitations caused by the numerous convolution and pooling operations in YOLOv8, YOLOv8x-pose often struggles to adapt to the variations in keypoint features at different scales in the image. Moreover, when the body parts of individuals are occluded, there is still scope for enhancing the precision of YOLOv8x-pose. Therefore, this paper proposes a human pose estimation model called  **CCAM-Person**. Based on YOLOv8x-pose and referencing the implementation framework of YOLO-pose^22^, our method simultaneously detects individuals and regress their keypoints in the image. Furthermore, we enhance the pose estimation performance by introducing additional receptive field expansion modules and visual attention mechanisms. We compare the optimized model with other state-of-the-art real-time HPE methods on the MS COCO 2017 dataset^23^ and the CrowdPose dataset^24^ to validate the efficacy of CCAM-Person in terms of real-time capability and regression accuracy.

Specifically, our contributions can be stated as:

- Firstly, CCAM-Person improves the backbone of the YOLOv8x-pose baseline. We replace the top-level C2F feature extraction block with a Multi-scale Receptive Field (${\textbf {MRF}}$) Module to alleviate the limitations of the model’s effective receptive region.
- Secondly, we enhance the feature fusion approach of YOLOv8 by introducing the Multi-path Feature Pyramid Network (${\textbf {MFPN}}$) instead of the original PANet. This further optimizes the interaction of information between different feature levels.
- Lastly, CCAM-Person incorporates the concept of Coordinate Attention (CA)^25^ and designs a novel Context Coordinate Attention Module (${\textbf {CCAM}}$) to enhance the precision of pose estimation through addressing issues caused by environmental noise or occluded body parts.

**Figure 2:**
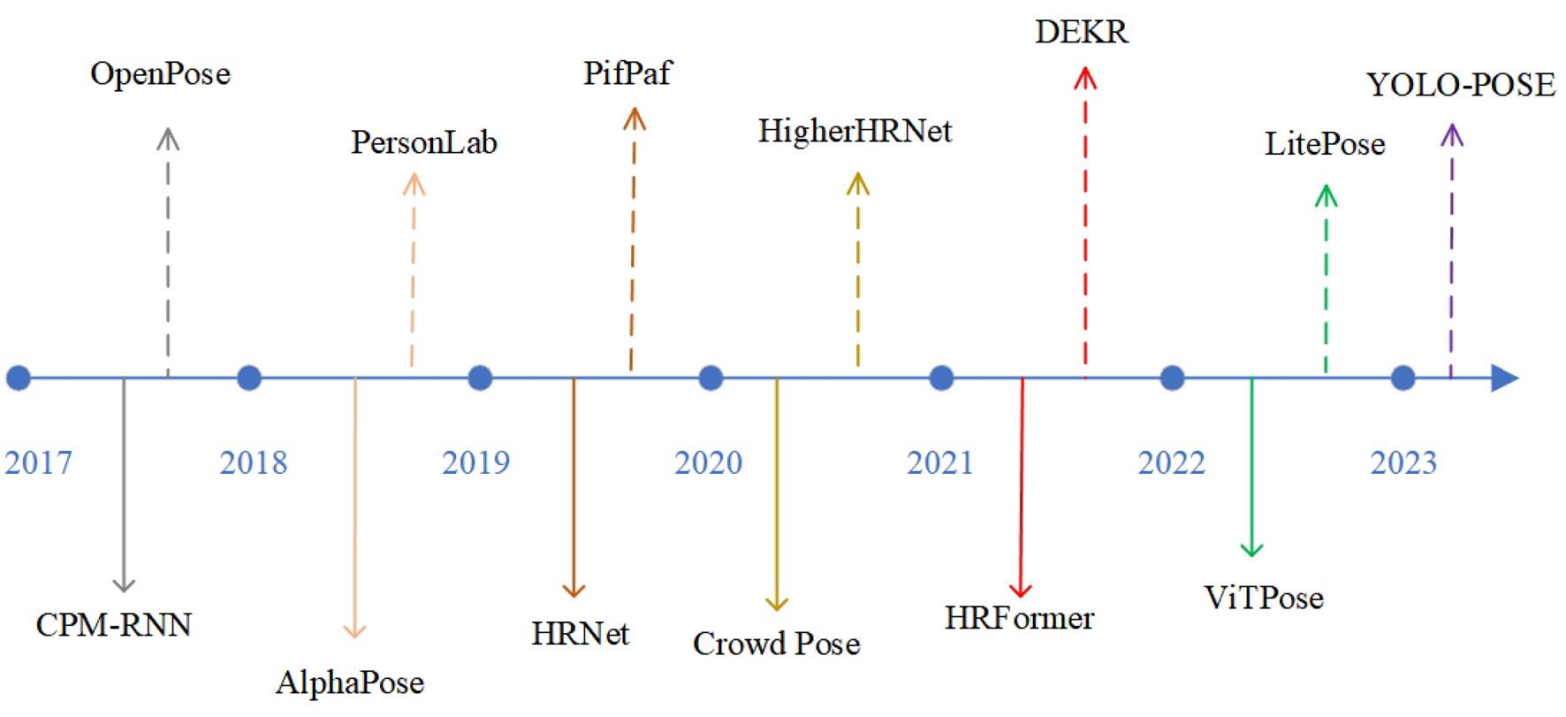
Overview of the development of HPE models.

Currently, mainstream strategies for MPE can be classified into two categories: single-stage and two-stage, as shown in Fig. 2. The two-stage approach initially employs object detectors or pedestrian detectors to detect human instances in the image. Then, for each detected human instance, a single-person pose estimation model is utilized for pose estimation. On the other hand, the single-stage approach identifies and connects the keypoints of human bodies through keypoint detection and association analysis. The single-stage strategy does not rely on the detection of human instances, allowing for simultaneous estimation of multiple people’s poses in a crowd. Each strategy has its advantages and disadvantages, with the single-stage strategy being widely adopted in real-time scenarios due to its high efficiency.

## Literature review

### Two-stage approach

To address issues related to inaccurate bounding box localization and redundant poses, Alphapose^26^ proposes a Region Multi-person Pose Estimation (RMPE) framework. Alphapose introduces a Symmetric Spatial Transformation Network (SSTN) to extract high-quality single-person regions and utilizes Parametric Pose Non-Maximum Suppression (Parametric Pose NMS) to eliminate redundant poses. However, it requires significant computational resources and algorithm optimization due to its high computational complexity. HRNet^27^ designs an efficient network architecture. Unlike previous methods that predict high-resolution heatmaps from low-resolution features, HRNet incorporates multiple parallel pathways with varying resolutions. High-resolution features are preserved, and features of various scales can be integrated with each other. This design integrates fine-grained low-level features with high-level semantic information, achieving more accurate regression. However, the network is relatively complex, and it presents challenges in terms of hyperparameter settings and adjustments.

Different from traditional CNN models, ViTPose^28^ adopts a novel Transformer architecture to map the input image to a fixed-length sequence and perform detection and recognition of human pose keypoints, achieving high-precision multi-person pose estimation. However, ViTPose has limited capability in handling local information and is not sensitive to the precise location of keypoints. Qiu et al.^29^ propose a new solution called DiffusionPose, which defines the 2D HPE problem as generating keypoint heatmaps from noisy heatmaps. Further improvement in the performance of DiffusionPose is achieved by introducing human structural prior information, making it one of the current cutting-edge techniques in terms of precision. However, the workflow of DiffusionPose is relatively complex, and it does not effectively address the overfitting issue of the Transformer architecture.

### Single-stage approach

OpenPose^30^ utilizes a convolutional neural network (CNN) to extract features from the image and regress all keypoints. It then connects these keypoints using graph algorithms and other post-processing operations to estimate the human pose. However, OpenPose is sensitive to image quality and lighting conditions, and it may be affected by background noise and interference. To address the challenge of significant pose estimation difficulty caused by scale variations, HigherHRNet^2^ employs a high-resolution feature pyramid to learn scale-aware representations. By incorporating multi-scale feature extraction and multi-level feature fusion, HigherHRNet augments the model’s resilience and precision in complex scenes and environments with significant variations. However, HigherHRNet requires a high-quality dataset with an extensive corpus of training data to fully learn pose patterns and relationships.

DEKR^3^ adopts a decoupled approach to regress the positions of human keypoints, transforming the pose estimation task into multiple independent keypoint regression problems. However, the decoupled regression method may face challenges in handling global consistency. Luo et al.^5^ propose the Self-Adaptive Heatmap Regression (SAHR) method and the Weighted Adaptive Heatmap Regression (WAHR) method to address challenges related to changes in human size and ambiguous human keypoint labels. Nevertheless, the implementation of these approaches is comparatively intricate and may not be optimal for exigencies that require real-time processing. LitePose^4^ achieves better performance and lower latency in edge device pose estimation tasks by utilizing a single-branch framework with large kernel convolutions. The inclusion of the Scale-Awareness module improves estimation accuracy, promoting advancements in real-time MPE. Nevertheless, there is still potential for enhancing the precision of regression.

### Broadening object detectors for keypoint estimation

In recent years, some models have employed the fundamental idea of object detection to build unified pose estimation regression frameworks, enabling simultaneous detection of human regions and regression of keypoints. Yang et al.^31^ propose Point-Set Anchors, which uses a set of anchor points for HPE. The method represents human pose as a set of point-set anchors and uses a neural network to detect and regress these anchors, thereby obtaining the human pose. However, this method relies on the selection of initial anchors, and different initial anchors may have an impact on the results. FCPose^32^ presents a fully convolutional multi-person pose estimation framework based on dynamic instance-aware convolutions. The keypoint estimation method with instance awareness eliminates ROI and post-processing operations, further enhancing the efficiency of multi-person pose estimation. However, when overlapping or occlusion occurs, it may lead to the failure of detecting certain keypoints, influencing the accuracy of pose estimation. DeepDarts^33^ formulates the HPE problem as a constrained optimization problem and incorporates contextual intelligence to enhance the precision of pose estimation. However, the constrained optimization method might converge to local optima in certain cases, affecting the overall accuracy of pose estimation. YOLO-Pose^22^ and KAPAO^34^ are the latest models in this field. They extend the latest real-time object detection methods by introducing additional human keypoint similarity loss (OKS)^35^, enabling the models to simultaneously detect human regions and keypoint positions.

## Research methodology

### Overview

To address the issues of inaccurate keypoint localization caused by limited receptive fields or loss of original features in existing real-time HPE methods, as well as the failure of pose estimation due to occlusion of body parts, we propose a real-time HPE model called CCAM-Person. Specifically, our method draws inspiration from the basic architecture of YOLOv8x-pose^22^. Building upon real-time object detection with YOLOv8, we additionally perform real-time regression of all human keypoints in the image, achieving simultaneous real-time region detection and pose estimation of individuals in the image.

We propose a real-time HPE model called CCAM-Person to address the limitations of existing methods, such as inaccurate keypoint localization due to limited receptive field or loss of original features, and pose estimation failure caused by occlusion. Specifically, our approach is inspired by the YOLOv8x-pose^36^ and extends the basic architecture of YOLOv8 for real-time object detection to simultaneously perform real-time regression on all human keypoints in the image, achieving both real-time region detection and pose estimation for people in the image.

**Figure 3:**
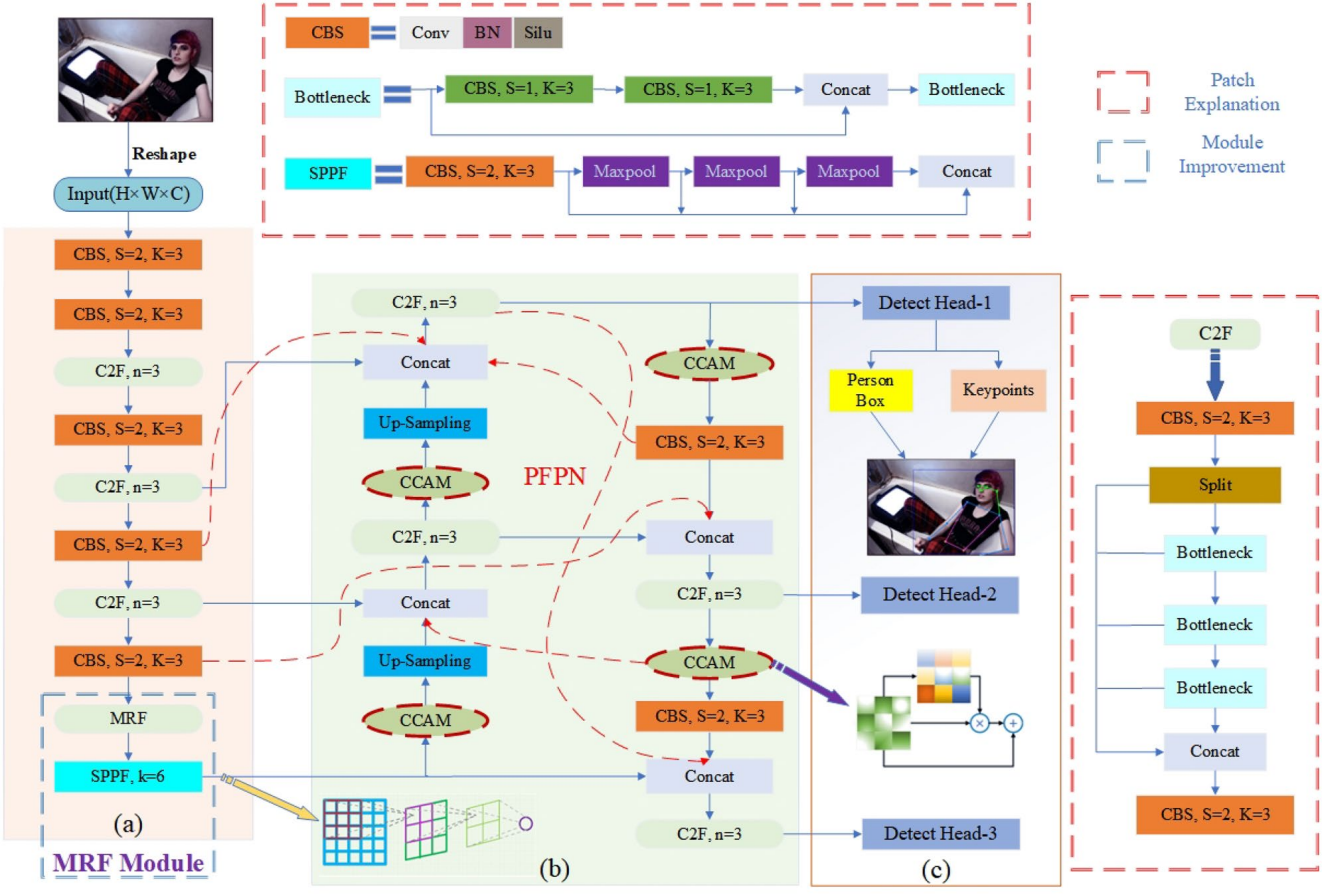
Holistic framework architecture of CCAM-Person. The backbone of the model, denoted as (**a**), adopts the design structure of CSPDarkNet-53 and introduces the CSPLayer_2Conv (C2F) convolution block. To improve the model’s receptive field, we introduce the MRF module in the deep feature block. The neck part, denoted as (**b**), replaces the original PANet feature fusion network with the MFPN to retain more original features at different resolutions. In addition, the CCAM is introduced to enhance the attention to important features. The head part, denoted as (**c**), follows the design of the decoupled heads in YOLOv8 and performs separate regression for both the human region and its keypoints to obtain the final human pose estimation results.

The CCAM-Person model utilizes the YOLOv8 detection algorithm to perform real-time localization and recognition of human targets in the image. At the same time, it applies a binary classification-like approach to detect all possible human keypoints in the image. Finally, a post-processing operation is used to match and group the keypoints with different ground-truth human bodies. The overall workflow of the model is illustrated in Fig. 3.

Our model borrows the architecture of YOLOv8x-pose and adopts a single-stage object detection approach to unify the modeling of human contours and keypoints, thereby achieving real-time pose estimation for people in the image. CCAM-Person mainly optimizes three aspects of the baseline: the feature representation of the backbone, the interaction and fusion of features in the neck part, and the learning of important feature cues, further improving the effectiveness of pose estimation.

In the end, the model generates a vector of length 6+3*n for each predicted bounding box. The first six values describe the position, type, and confidence of the human region, while the subsequent 3*n values represent the position and confidence of each keypoint. This process can be summarized as follows:

1 $$\begin{aligned} {S} = \left\{ {B_x},{B_y},W,H,bo{x_{conf}},clas{s_{conf}},P_x^1,P_y^1,P_{conf}^1,\ldots ,P_x^n,P_y^n,P_{conf}^n\right\} \end{aligned}$$

In the aforementioned equation, S represents the set of elements that we obtain as the final prediction of our task. The first six elements in the set describe the relevant information about the bounding box of the person in the image. Specifically, $\text {B}_{x}$ and $\text {B}_{y}$ denote the coordinates of the bounding box’s center point, while W and H depict its width and height, respectively. Together, these four elements encompass the positional characteristics of the individual. Furthermore, $\text {box}_{conf}$ and $\text {class}_{conf}$ respectively represent the confidence parameters of the bounding box and the probability of it containing an individual. As for $\text {P}_{x}^{1}$, $\text {P}_{y}^{1}$, $\text {P}_{conf}^{1}$,..., $\text {P}_{x}^{n}$, $\text {P}_{y}^{n}$, and $\text {P}_{conf}^{n}$, they individually represent the horizontal and vertical coordinates, as well as the corresponding confidence levels, of the total n key points associated with the individual.

### Multi-scale receptive field module

To better help the model understand the various sizes of people in the image and improve the accuracy of subsequent regression and classification tasks, we have integrated the MRF module into the Backbone part of the model.

Generally, shallow feature layers in the network contain more detailed or textural features, while deep feature layers contain more global or semantic features. For multi-scale HPE tasks, receptive field and resolution are key factors. Deep feature layers have smaller feature map resolutions and cover larger receptive fields per unit. However, they often lack detailed texture information from lower layers, which can lead to a decrease in prediction accuracy. To alleviate this issue, we use the MRF module to replace the original final C2f convolution block in the Backbone to aggregate more low-level features.

**Figure 4:**
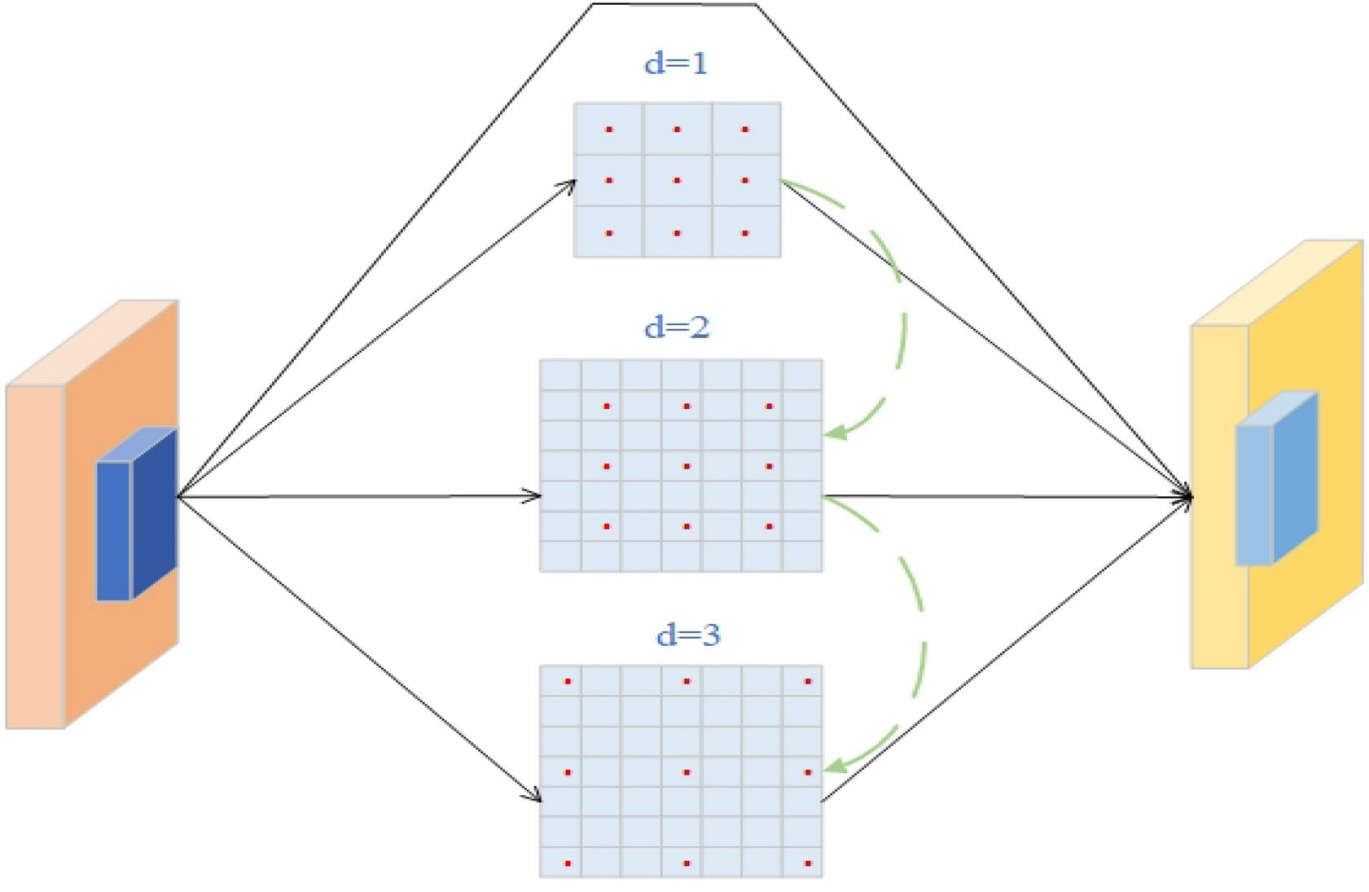
Fusion of deep-level features in the backbone using dilated convolutions.

Specifically, we roughly describe the hierarchy of the model’s Backbone as {C1, C2, C3, C4, C5}, with C1 defined as the initial layer and C2 defined as the detail layer. We can adopt the idea from TridentNet^37^ to fuse the information from C1 and C2 with C5 using dilated convolutions^38^, as shown in Fig. 4.

**Figure 5:**
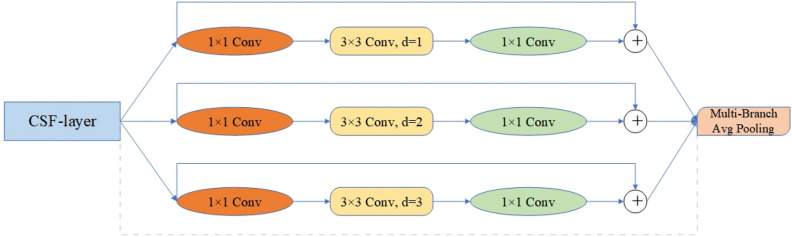
Internal structure of the MRF module. We replace the top-level C2F feature layer in the original backbone with MRF. MRF consists of $\text{1 }\times 1$ convolution, $\text{3 }\times 3$ convolution with different dilation rates, and a multi-branch fusion pooling layer.

Differences in receptive fields often indicate different abilities to capture long-range dependencies. Simply fusing low-level information may lead to a decrease in detection accuracy for large and medium-sized people in the image. Our MRF module draws inspiration from the concept of DeepLab^39^ and achieves downsampling of shallow features (C1, C2) by dynamically adjusting the dilation rate (d). Subsequently, we fuse the features from three branches with different dilation rates (C1, C2, C5) to facilitate information interaction across different receptive fields. Each branch is trained with its own weights to adapt to different image samples and fully utilize information at different resolutions. Weighted operations are also utilized to balance the contribution of different branches. The internal structure of MRF is illustrated in Fig. 5.

### Multi-path feature pyramid network

To better leverage the important feature information extracted by the Backbone network in the previous stage, we have designed the MFPN based on the ideas of UNet3+^40^ and AFPN^41^ for more efficient information interaction between different feature levels, aiming to enhance the accuracy of pose estimation in the CCAM-Person model.

The YOLOv8x-pose human pose estimation model inherits the basic idea of feature fusion module from YOLOv5x^42^ in the neck part and uses the Path Aggregation Network (PANet)^18^ as the processing module for the Backbone features. PANet employs both top-down and bottom-up information propagation paths: the top-down path transfers high-level semantic features to low-level features through upsampling, and the bottom-up path extracts detailed information from low-level features to enrich high-level features. However, the pyramid structure based on PANet still has some limitations. Firstly, the scaling operations on the feature maps inevitably result in feature loss, leading to the lack of semantic or detailed information in the final output feature map. Secondly, for the HPE task, the useful features must contain detailed or semantic information about human keypoints. The high-level or low-level features in PANet need to interact with features at different scales through multiple intermediate scales before being fused with bottom-level or top-level features. In this propagation and interaction process, the high-level semantics or low-level details may be lost or degraded.

**Figure 6:**
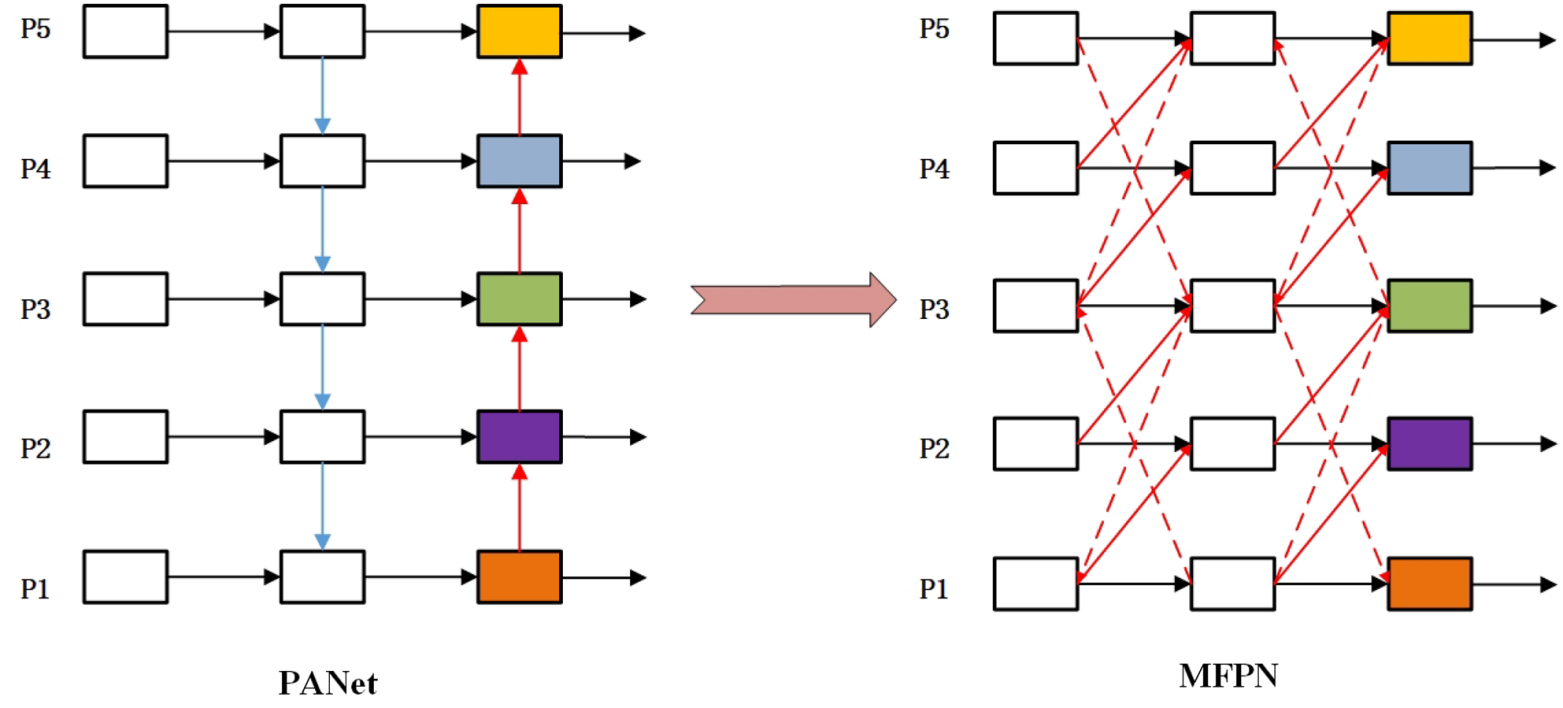
Difference in feature exchange Pyramid between PANet and MFPN.

To address this, we introduce the MFPN structure in the Neck part to replace the PANet structure in the original YOLOv8x-pose, further optimizing the multi-scale feature fusion. The comparison of information interaction between the two feature pyramids is shown in Fig. 6.

Compared with PANet, our MFPN allows the feature maps to incorporate more feature information from different receptive fields. Specifically, PANet only uses progressive top-down or bottom-up feature fusion paths, while we add some cross-layer feature fusion pathways on top of that. The advantage of this approach is that deep-level features can better receive detailed information from shallow-level features, while shallow-level features can more directly receive semantic information from deep-level features, reducing information loss during propagation. To better illustrate the processing of features between different levels, we take the example of the information flow in the P3 feature layer. Under the feature fusion mode of PANet, the calculation of information can be represented as:

2 $$\begin{aligned} P_3^{\mathrm{{out}}} = Conv(P_3^{in} + Resize(P_4^{in}) + Resize(P_2^{out})) \end{aligned}$$

where $\text {P}_{3}^{in}$ represents the input feature map of the third stage with a depth of the feature layer, and $\text {P}_{3}^{out}$ represents the output feature map of the third stage. Based on our MFPN information fusion mode, the composition of the P3 layer feature can be approximated as:

3 $$\begin{aligned} \begin{aligned} P_3^{\mathrm{{out}}} =&Conv\left( {\lambda _1}P_3^{in} + {\lambda _2}P_2^{in} + {\lambda _3}Resize(P_5^{in})\right. \\&\left. + {\lambda _4}Resize(P_2^{out}) + {\lambda _5}Resize(P_1^{in}) + {\lambda _6}P_2^{mid}\right) \end{aligned} \end{aligned}$$

where ${\lambda }_{i}$ represents the weight ratio of different information flows, which will be adaptively adjusted during model training, and $\text {P}_{3}^{mid}$ represents the intermediate temporary feature map generated by the P2 layer.

The weight parameters (${\lambda }_{1}$,..., ${\lambda }_{6}$) in the MFPN represent different aspects of features. Benefiting from the ability of the YOLO algorithm to process the entire image in one pass, keypoint regression tends to be more accurate when dealing with larger-sized individuals. However, it often performs poorly in scenarios with dense crowds or smaller-sized individuals. Therefore, our MFPN effectively combines features from different depths through multi-path connections. MFPN enables the organic integration of high-level semantic information with low-level detailed texture information, further improving the effectiveness of pose estimation.

Our MFPN Neck module, through the use of cross-layer multi-path feature fusion mode, achieves a more comprehensive combination of features at different levels. Meanwhile, the Head can receive more detailed texture information from lower layers and semantic information from higher layers, preserving a greater variety of original features. This has a positive effect on keypoint regression for people of different scales in the image.

### Context coordinate attention module

The visual attention mechanism refers to the ability to focus on specific parts of an object while ignoring irrelevant surrounding information, enhancing object recognition and understanding. This mechanism plays a crucial role in the task of HPE, especially when occlusion occurs on certain keypoints of the human body. Visual attention helps concentrate attention on the key areas of the human body, enabling more accurate regression of the keypoints.

Common visual attention mechanisms^43–46^, such as channel attention and spatial attention, typically model feature information from either channel or spatial dimensions. Channel attention^43,44^ assigns weights to different feature channels and allocates adaptive weights to each channel. However, it models spatial information poorly and lacks sensitivity to the positions of the human keypoints. On the other hand, spatial attention^45,46^ helps improve the regression of spatial information related to human pose. However, relying solely on spatial attention can be prone to image noise and increase errors. Based on the work of CA^25^, we propose a novel CCAM to model the channel-spatial mixed domain of image features.

**Figure 7:**
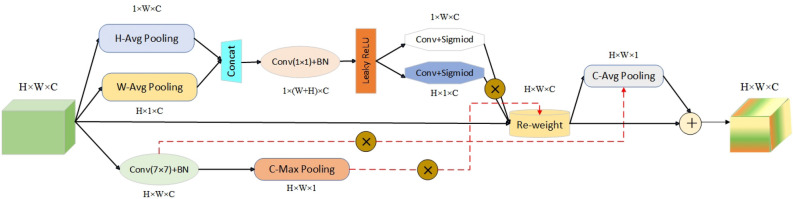
Internal implementation structure of the context coordinate attention module.

CCAM models different positions in the feature space along the width and height dimensions of the image. Additionally, we design additional context-aware pathways to propagate spatial feature response signals with different receptive fields for each position. This signal can be used to approximate the rough location of all human keypoints in the module’s image. This approach enables more accurate regression of the human keypoints, addressing the loss of pose regression accuracy caused by partial occlusion. The internal implementation structure of CCAM is illustrated in Fig. 7.

Specifically, the implementation process of CCAM can be summarized as follows:

The first stage of attention modeling primarily focuses on different positions in the feature space. Firstly, the feature block X extracted by the backbone network is subjected to global max-pooling operations along the W and H dimensions to compress information in different dimensions. Global max-pooling along the W and H directions generates feature maps of size $\text{ H }\times 1\times C$ and $\text{1 }\times W\times C$, respectively. This operation avoids compressing all feature information into a single dimension. Simultaneously, a large kernel convolution ($\text{7 }\times 7$) is applied to preserve more semantic information from a larger receptive field. The mathematical representation of this process is shown in the equation below:

4 $$\begin{aligned} z_c^h(H)&= \frac{1}{W}\sum \limits _{0 \le i \le W} {{X_c}(h,i)} \end{aligned}$$

5 $$\begin{aligned} z_c^w(W)&= \frac{1}{H}\sum \limits _{0 \le j \le H} {{X_c}} (j,w) \end{aligned}$$

6 $$\begin{aligned} X'&= Batch\_Normalization[Con{v_{7 \times 7}}(X)] \end{aligned}$$

Here ${\textbf {z}}_{c}^{w}(H)$ and ${\textbf {z}}_{c}^{w}(W)$ represent the global max-pooling operations along the W and H dimensions on the grouped feature maps. Afterwards, the processed features are concatenated and passed through a shared $\text{1 }\times 1$ convolutional transformation function $T_1$, followed by an activation operation, resulting in a feature map of size $\text{1 }\times (W+H) \times C$. The specific operation is as follows:

7 $$\begin{aligned} f = \delta \left( {T_1}\left( {z^h},{z^w}\right) \right) \end{aligned}$$

In the equation, $~\sigma$ represents the non-linear activation operation, and leaky ReLU is used in this case. f represents the feature map obtained after the previous stage processing. Next, the feature map f is split along the W dimension into two independent tensors, $\text {f}^{w}\in \mathbb {R}^{1\times W\times C}$ and $\text {f}^{h}\in \mathbb {R}^{H\times 1\times C}$, which are aligned in dimension using a $1\times 1$ convolution. In the next stage, we normalize (sigmoid) the feature tensors to obtain two attention vectors: $\text {g}^{h}\in \mathbb {R}^{H\times 1\times C}$ and $\text {g}^{w}\in \mathbb {R}^{W\times 1\times C}$. The attention distribution based on different positions is calculated using matrix multiplication. The specific operation is as follows:

8 $$\begin{aligned} {g^h}&= \sigma \left( {F_h}\left( {f^h}\right) \right) \end{aligned}$$

9 $$\begin{aligned} {g^w}&= \sigma \left( {F_w}\left( {f^w}\right) \right) \end{aligned}$$

10 $$\begin{aligned} Attentio{n_{coordinate}}&= {g^w} \otimes {g^h} \end{aligned}$$

The second stage of attention modeling mainly integrates spatial information from different receptive fields. Firstly, we compress the attention obtained in the previous stage along the channel dimension using average pooling to obtain $\text {S}_{1}\in \mathbb {R}^{H\times W\times 1}$. Similarly, we compress the feature X’ processed by the large kernel convolution along the channel dimension using max pooling to obtain $\text {S}_{2}\in \mathbb {R}^{H\times W\times 1}$. Next, we combine the attention distributions from different receptive fields using the matmul operation:

11 $$\begin{aligned} {U_1}&= {S_2} \otimes Attentio{n_{coordinate}} \end{aligned}$$

12 $$\begin{aligned} {U_2}&= X' \otimes {S_1} \end{aligned}$$

Finally, we aggregate the pairwise results to form the final attention weights and propagate them to the original feature space:

13 $$\begin{aligned} Output(i,j) = X(i,j) \otimes ({U_1} \oplus {U_2}) \end{aligned}$$

In summary, compared to the traditional coordinate attention mechanism, our CCAM preserves more spatial features from different receptive fields by incorporating secondary spatial modeling on the feature block. Moreover, by simulating the distribution of human keypoints in the image through spatial modeling, we provide spatial strength signals to the coordinate attention mechanism, further enhancing the regression of human keypoints. We provide the implementation of CCAM in pseudocode form, as shown in $~{\textbf {Algorithm.}}$ 1.

 Our CCAM attention mechanism bears resemblance to certain attention structures previously proposed in the field of image semantic segmentation^47,48^. However, there are inherent differences to be noted. Semantic Aware Channel Selection (SACS)^47^ incorporates a semantic encoding process on top of the original channel attention mechanism, enhancing the model’s response to crucial semantic feature signals in the channel dimension. Similarly, the Squeeze-and-Attention (SA) module^48^ optimizes the SENet^43^ with a deeper level of refinement. Unlike SENet, SA employs average pooling to downscale the feature maps without fully squeezing them to $\text{1 }\times 1\times C$. This allows SA to retain certain spatial features, enabling the aggregation of non-local features and improving the effectiveness of semantic segmentation.

 Both of the aforementioned attention mechanisms primarily focus on deep-level modeling of feature channels. In contrast, our CCAM attention mechanism takes a channel-spatial hybrid modeling approach. CCAM utilizes a dual-branch framework: the first branch, inspired by the concept of CA^25^, performs weight modeling on each position within the feature block. As human keypoints are often distributed unevenly in images, in the second branch, we employ the notion of spatial attention to model the weights of different positions in the image. The final attention response is obtained by merging the weights from both branches.

 The CCAM attention mechanism provides a more comprehensive modeling of both channel and spatial domains. This enhances the ability of our model to capture important semantic information and spatial relationships, thereby improving the accuracy of human pose estimation.

**Algorithm 1:**
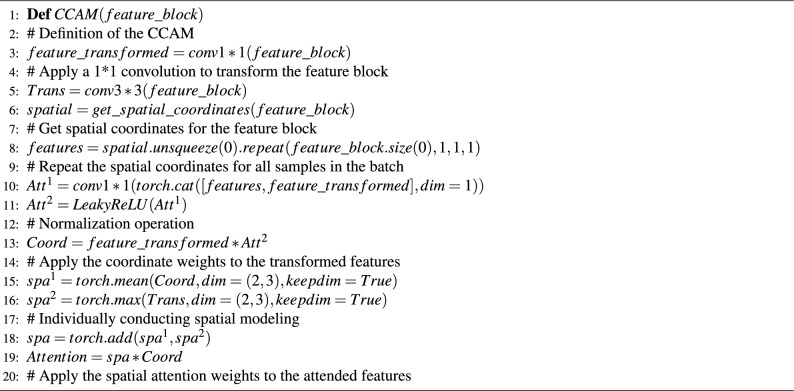
PyTorch-like Code for Context Coordinate Attention Module

## Experiments and analyses

In this section, we conducted a fair comparison between our proposed CCAM-Person model and recent real-time HPE methods on the MS COCO 2017 keypoint challenge dataset and the CrowdPose multi-person pose estimation dataset. Additionally, we conducted corresponding ablation experiments to validate the rationality of our module design.

### Experimental setting

The training process of our model was performed using NVIDIA GeForce RTX 3080 Ti GPUs on the Ubuntu 18.04 LTS operating system. We utilized the PyTorch deep learning framework with GPU acceleration using NVIDIA CUDA.

#### Datasets

The MS COCO 2017 dataset is extensively employed for evaluating and comparing the performance and accuracy of various pose estimation algorithms in the field of HPE. This dataset consists of over 20,000 images, each annotated with keypoints corresponding to 17 body joints, including the head, neck, shoulders, elbows, wrists, hips, knees, and ankles. Each keypoint is represented by its pixel coordinates. The dataset also provides bounding box annotations for each person instance to locate the human regions. The images in the dataset are captured from real-world scenarios, covering various environments and activities, including indoor and outdoor settings, as well as single and multiple individuals.
(2) The CrowdPose dataset is a large-scale dataset for crowd pose estimation. It comprises thousands of crowd images captured in real-world scenes, covering various common crowd activities and scenarios. The dataset is characterized by diversity, scale, and density. In terms of diversity, the dataset includes a variety of crowd activities and scenes, captured at different angles and distances. The dataset is large in scale, providing rich training and testing samples with thousands of crowd images. Moreover, the CrowdPose dataset exhibits high crowd density, with some images containing a large number of people and complex occlusions and overlaps. The dataset provides annotations for the keypoints of each person in the crowd, including keypoints for the head, arms, legs, and other body parts. The keypoint annotations have undergone careful manual verification to ensure accuracy and precision.

#### Evaluation metrics

In the task of HPE, the Average Precision (AP) metric based on OKS is widely used to evaluate algorithm performance. The AP metric calculates the average precision based on different thresholds and object sizes. For example, AP50 represents the average precision at an OKS threshold of 50, while AP75 represents the average precision at an OKS threshold of 75.

To assess the performance of pose estimation for persons of different scales, the COCO dataset provides additional metrics. $\text{ A }P^M$ (AP for medium objects: $\text{3 }2^2<area<96^2$) represents the average precision for medium-sized objects, and $\text{ A }P^L$ (AP for large objects: $\text{ a }rea>96^2$) represents the average precision for larger objects. These metrics consider the different object sizes comprehensively, enabling a more comprehensive evaluation of algorithm performance.

To better measure the model’s performance in different application scenarios, CrowdPose introduces the concept of the crowd index and divides the images into three categories based on the range of crowd index values: easy (0–0.1), medium [0.1–0.8), and hard [0.8–1), corresponding to $\text{ A }P^E$, $\text{ A }P^M$, and $\text{ A }P^H$, respectively. This graded evaluation takes into account the challenges posed by crowd density in pose estimation and provides a more accurate performance evaluation.

In addition, the Average Recall (AR) metric is used to measure the recall performance of the algorithm, evaluating the coverage range of pose estimation. The recall rate reflects the model’s ability to recognize each keypoint and can assess overall performance.

Finally, the Latency (ms) metric reflects the inference time of the model, including the time required for model forward propagation and post-processing. This metric facilitates the evaluation of the algorithm’s real-time performance and applicability.

#### Training

In the preparation stage, we employed data augmentation strategies including Mosaic^49^ and Cutout^50^. The input images were resized to $1280\times 1280$ and necessary padding was applied. Instead of traditional Adam, we used the recently released Lion^51^ optimizer. The maximum number of training epochs was set to 300, with an initial learning rate of $~\text{5 }e^{-4}$, which was decreased to $~\text{5 }e^{-5}$ at the 200th epoch. At the 240th epoch, the learning rate was further reduced to $~\text{ e}^{-5}$. To ensure training stability and efficiency, the minimum batch size was set to 20.

**Figure 8:**
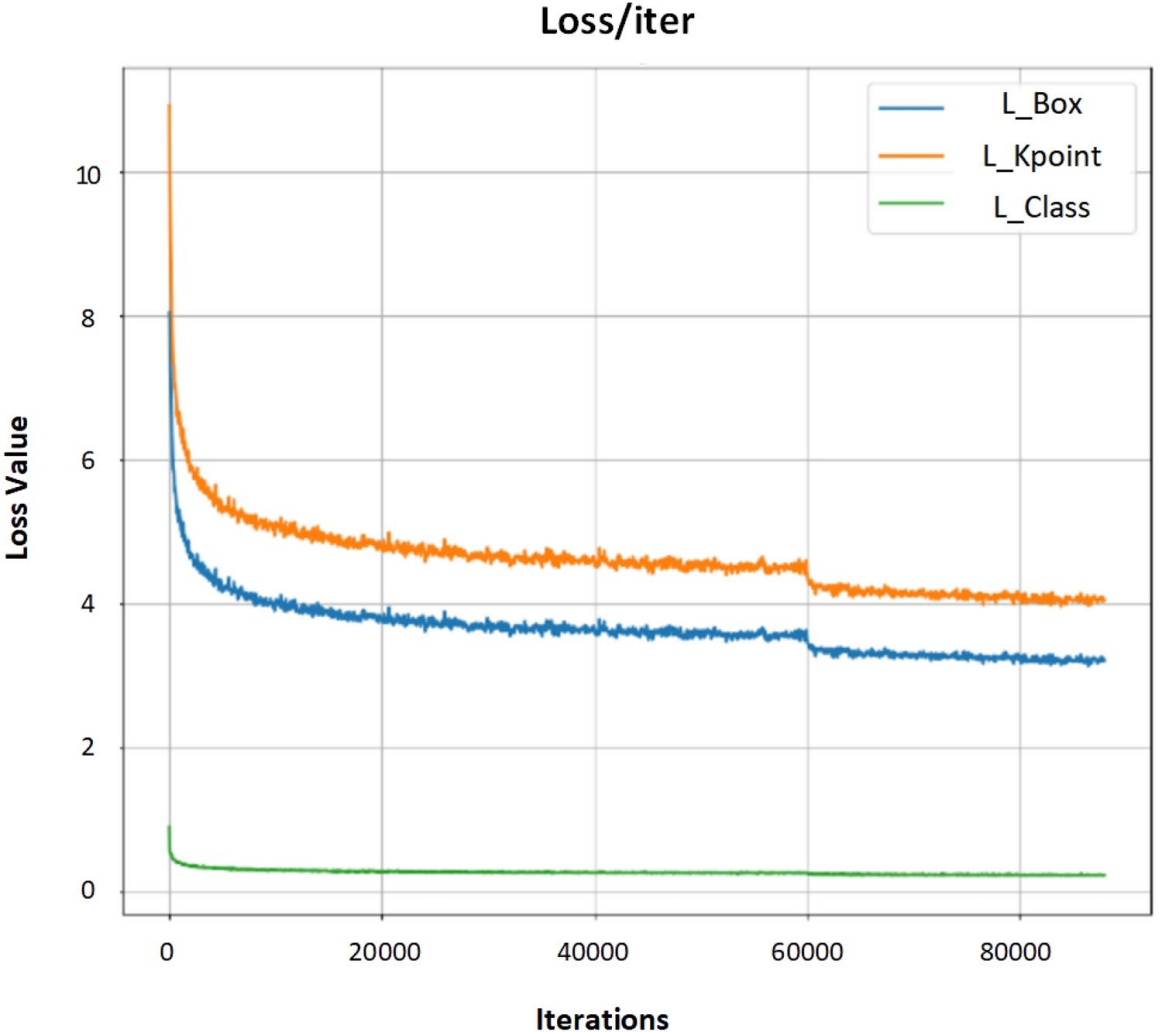
The trend of changes in each component’s loss function during the model training process with respect to the number of iterations.

The loss function of the model primarily comprises three constituents: classification loss ($~\text{ L}_{cls}$), bounding box regression loss ($~\text{ L}_{box}$), and human keypoint loss ($~\text{ L}_{kpoints}$). The changes in the loss functions during model training are illustrated in Fig. 8. The overall loss function for the task can be roughly represented as:

14 $$\begin{aligned} {{{\mathscr {L}}}_{total}} = \sum \limits _S {({\lambda _{cls}}{{\mathscr {L}}_{cls}} + {\lambda _{box}}{{{\mathscr {L}}}_{box}} + {\lambda _{kpo{\textrm{int}} }}{{{\mathscr {L}}}_{kpo{\textrm{int}} }})} \end{aligned}$$

### Experimental results on publicly available datasets

Our CCAM-Person HPE model is based on the YOLOv8 framework and draws inspiration from the keypoint regression approach of YOLO-POSE, enabling simultaneous detection and pose estimation of all individuals in an image. Furthermore, we have improved the Backbone and Neck components of the YOLOv8 model to further enhance the accuracy of HPE. As shown in Table 1, we have conducted a fair comparison with other recent real-time HPE methods on the MS COCO 2017 dataset.

**Table 1:** Comparison with real-time HPE methods on COCO keypoint 2017 test-dev set.

Method	Input size	AP	${AP}^{50}$	${AP}^{75}$	${AP}^{M}$	${AP}^{L}$	AR	Latency (ms)
OpenPose^30^	960	61.8	84.9	67.5	57.1	68.2	66.5	366
HGG^52^	960	67.6	85.1	73.7	62.7	74.6	71.3	–
HigherHRNet-W32^2^	960	66.4	87.5	72.8	61.8	74.2	73.8	1653
HigherHRNet-W48^2^	960	70.5	89.3	77.2	66.6	75.8	74.9	1890
DEKR-W32^3^	960	67.3	87.9	74.1	64.8	75.1	73.2	1441
DEKR-W48^3^	960	71.0	89.2	77.3	67.1	76.9	76.7	2237
CenterGroup-W48^53^	960	71.1	90.5	77.5	66.9	76.7	77.1	2196
FCPose^32^	960	65.6	87.9	72.6	62.1	72.3	72.6	188
YOLOv5l6-pose^22^	960	68.5	90.3	74.8	66.8	76.5	75.0	132
KAPAO-L^34^	960	70.3	91.1	77.8	66.3	76.8	77.7	163
PRTR^54^	960	72.1	90.4	79.6	68.1	79.0	79.4	96
HRFormer^55^	960	74.4	92.2	82.3	70.7	80.5	79.8	147
ViTPose-B^28^	960	74.7	92.8	**82.6**	71.0	80.6	80.2	122
YOLOv8x-pose^36^	960	72.1	91.5	78.4	67.2	78.3	77.9	**78**
**CCAM-Person**	960	**74.9**	**93.7**	80.8	**69.1**	**81.4**	**82.1**	110

Significant values are in bold.

The experimental outcomes explicated below suggest that CCAM-Person achieves an estimation accuracy of 74.9% on the COCO 2017 test set. Compared to other popular real-time HPE methods^2,3,22,34^, our model demonstrates competitive results in all metrics. Benefiting from our improvements, CCAM-Person achieves a 2.8% improvement in accuracy and a 4.2% improvement in recall rate compared to the baseline. In comparison to methods based on Vision Transformers^28,54,55^, our approach still exhibits certain advantages in terms of both accuracy and speed. Although the introduction of attention modules and multi-path fusion modes leads to a certain decrease in inference speed, even when resizing the input image to $960\times 960$, CCAM-Person still maintains a competitive processing rate, meeting the temporal constraints of a majority of tasks.

**Table 2:** Results obtained on the CrowdPose test-dev set.

Method	AP	${AP}^{50}$	${AP}^{75}$	${AP}^{E}$	${AP}^{M}$	${AP}^{H}$	AR	Latency(ms)
OpenPose^30^	48.0	61.5	53.7	62.7	48.7	32.3	53.2	368
HigherHRNet-W48^2^	67.6	87.4	72.6	75.8	68.1	58.9	73.1	1650
DEKR-W48^3^	68.0	85.5	73.4	76.6	68.8	58.4	73.6	2235
CenterGroup-W48^53^	70.0	89.7	75.7	77.3	70.8	63.2	76.1	2195
YOLOv5l6-pose^22^	67.1	87.1	72.2	75.1	67.6	59.1	74.5	131
KAPAO-L^34^	68.9	89.4	75.6	76.6	69.9	59.5	75.7	165
HRFormer^55^	72.6	85.4	76.7	76.6	73.5	59.5	76.1	148
ViTPose-B^28^	74.2	85.1	**78.9**	79.8	**75.9**	65.3	77.3	122
YOLOv8x-pose^36^	70.9	90.8	75.5	78.6	72.2	62.2	76.7	**78**
**CCAM-Person**	**74.4**	**92.7**	78.4	**80.4**	75.7	**66.9**	**80.2**	111

Significant values are in bold.

The empirical findings in Table 2 illustrate that CCAM-Person exhibits high accuracy and an acceptable running speed on the CrowdPose keypoint detection dataset. Compared to the original YOLOv8x-pose model, our approach achieves a 3.5% increase in AP. Interestingly, our method achieves a notable improvement of 4.7% in the ${\textbf {AP}}^{H}$ metric, indicating that our improvements result in better pose estimation performance in densely crowded scenes.

### Ablation experimental study

In order to independently verify the efficacy of various proposed enhancement modules, we performed ablation experiments in this section to meticulously assess the functionalities of distinct modules.

#### Baseline architecture ablation experiment

These experiments examined the effect of enhancements made to the model’s Backbone (MRF) and Neck (MFPN) on the precision of HPE tasks. The evaluation of the baseline model’s performance, before and after the improvements, was carried out using the AP and AR metrics. The experimental outcomes, concerning the COCO val2017 dataset, are presented in Table 3.

**Table 3:** Baseline enhancement effects.

Method	Backbone		Neck		AP	AR	Latency (ms)
	C2F	C2F+MRF	PANet	MFPN			
CCAM-Person	$\checkmark$		$\checkmark$		73.1	77.8	**96**
	$\checkmark$			$\checkmark$	73.8	79.5	99
		$\checkmark$	$\checkmark$		74.2	80.4	108
		$\checkmark$		$\checkmark$	**74.7**	**81.9**	110

Significant values are in bold.

The results of the ablation experiments demonstrate that our improvements to the baseline in the Backbone and Neck components lead to an improvement of approximately 1.6% in estimation accuracy. Additionally, our baseline design introduces more information from different receptive fields, enabling the detection of more keypoints in the image, resulting in a 4.1% increase in recall rate.Despite the increased complexity introduced by the MRF module, resulting in a slight decrease in the inference speed of the model, the inference latency of around 110 ms remains acceptable for real-time HPE tasks.

#### Attention module ablation experiment

In this part of the experiment, we explored the impact of introducing CCAM on the overall performance of the model. We compared the accuracy before and after the introduction of the attention mechanism. Furthermore, we compared CCAM with several popular visual attention mechanisms, including SENet^43^, CBAM^45^, and ECA^44^, to validate the advantages of our proposed improved attention module in the pose estimation task. The experimental results on the COCO val2017 dataset are shown in Table 4.

**Table 4:** Ablation study on attention modules.

Method	AP	${AP}^{50}$
Baseline	73.2	91.5
Baseline &SENet	73.7	91.7
Baseline &CBAM	74.4	92.2
Baseline &ECA	74.0	92.3
**Baseline &CCAM**	**74.7**	**93.6**

Significant values are in bold.

**Figure 9:**
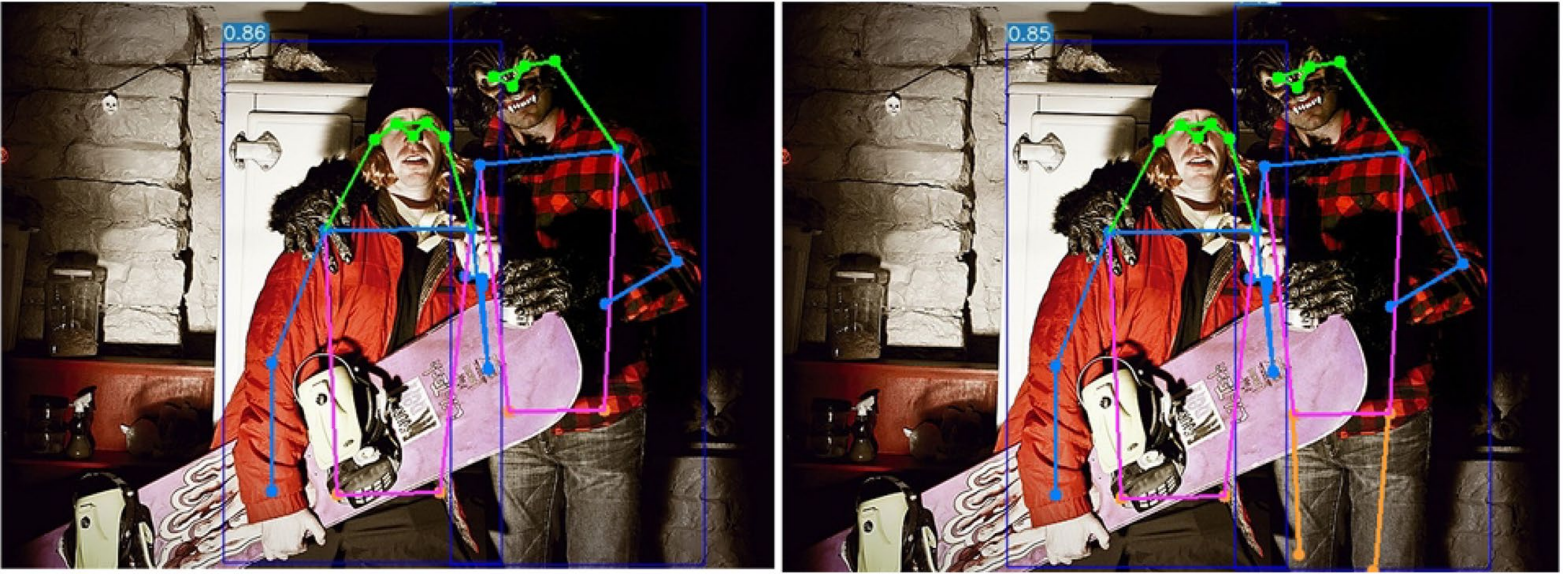
Differences in the efficacy of anterior-posterior human pose estimation with the inclusion of CCAM.

The above results indicate that the introduction of visual attention mechanisms can effectively enhance the model’s focus on important information, thereby improving the pose estimation performance. The introduction of CCAM allows the model to better focus on important positions within feature blocks and process specific feature information more accurately. The introduction of CCAM results in a 1.5% improvement in AP and a 2.1% improvement in $\text {AP}^{50}$, showing better accuracy improvement compared to other common visual attention mechanisms. Furthermore, the introduction of CCAM can also mitigate the failure of keypoint regression due to occlusion to a certain extent, enabling more keypoints to be accurately regressed, as shown in Fig. 9.

### Visual analytics

**Figure 10:**
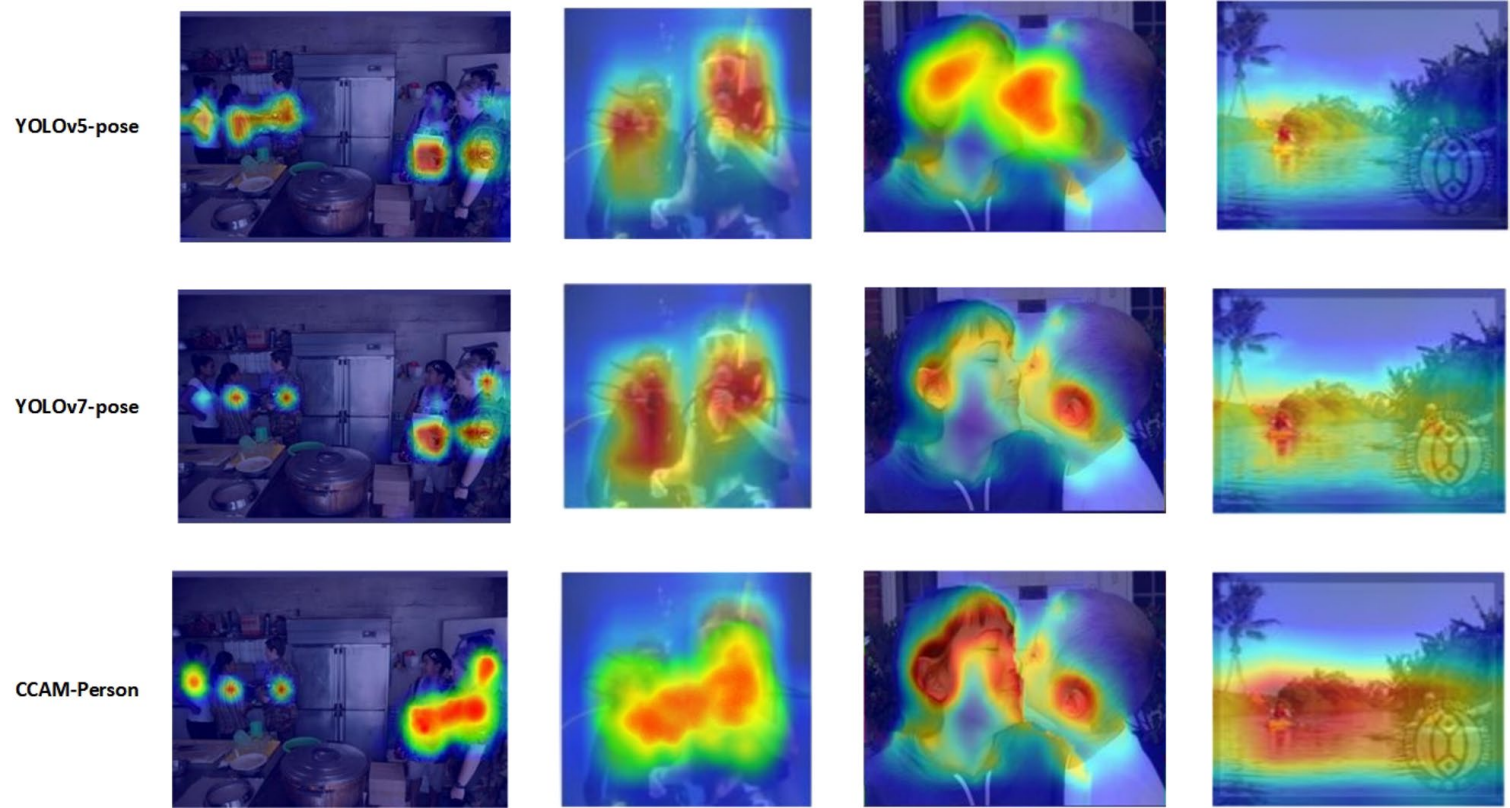
Grad-CAM analysis for partial real-time HPE model.

Grad-Cam++^56^ is a gradient-based class activation mapping method widely used in deep learning-based computer vision tasks such as object detection, image classification, and object localization. This method visualizes the activation levels of different feature maps in a neural network, helping to understand and explain the basis for network decisions, and providing an interpretable and visually intuitive analysis tool. Grad-Cam++ utilizes a dual principle, leveraging positive and negative gradient information to more accurately capture key regions, resulting in more accurate and discriminative class activation maps. We applied Grad-CAM++ to visualize several different real-time HPE models, and the heatmap results shown in Fig. 10 reflect their focus on different regions.

**Figure 11:**
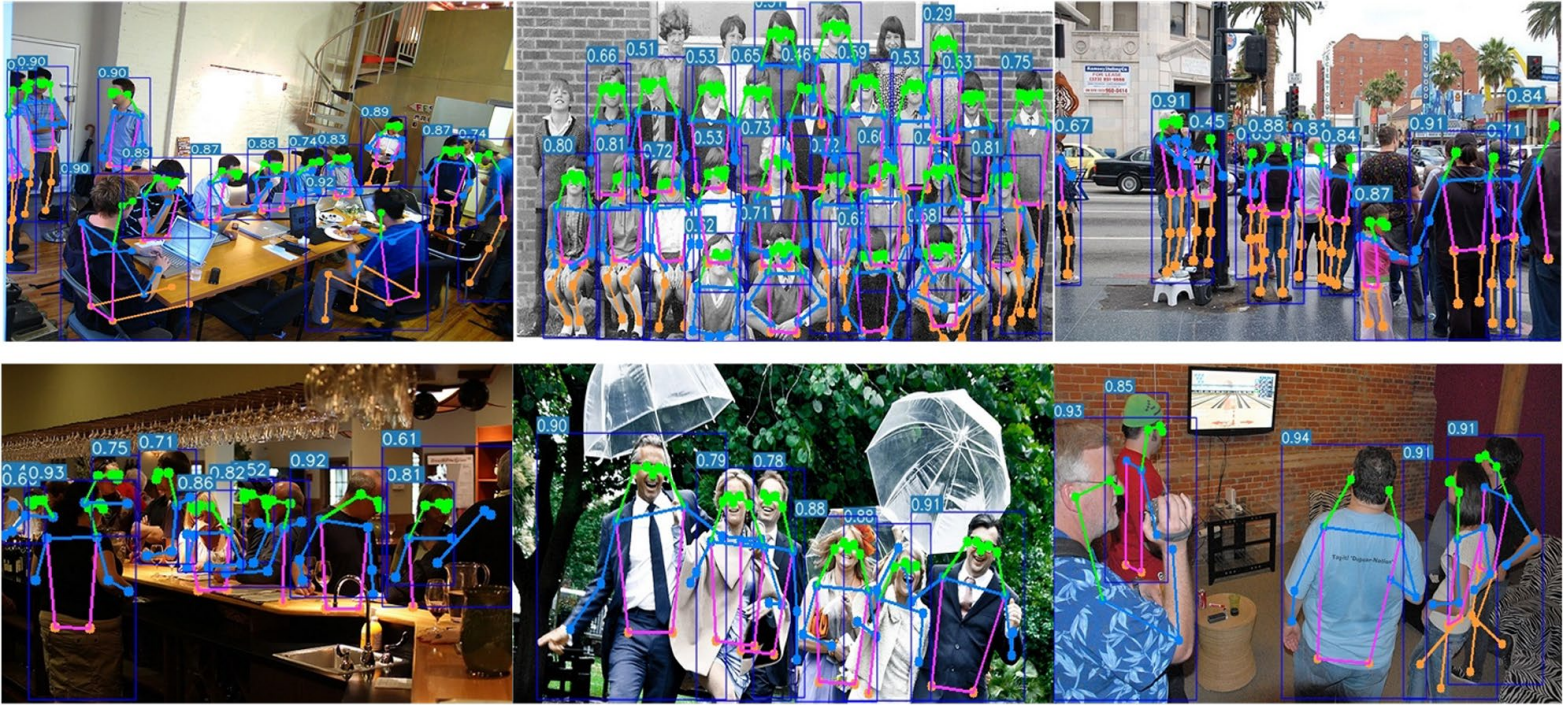
Visualization results for CCAM-Person.

The results of the above Class Activation Mapping demonstrate that compared to some other real-time HPE methods, our model pays higher attention to the person regions in the image. At the same time, CCAM-Person exhibits lower attention to the image background, reducing the interference of background noise on pose estimation. These factors contribute to the performance improvement of our method. The HPE outcomes, utilizing the CCAM-Person model, are depicted in Fig. 11.

## Discussion

Our proposed CCAM-Person model builds upon the YOLOv8x-pose framework while incorporating the keypoint regression idea from the YOLO-Pose model. The stacking of downsampling operations in the backbone of the YOLOv8x-pose model leads to feature information loss, and the PANet in the Neck component is limited in establishing effective feature fusion due to receptive field constraints. To address the deficiencies in the baseline mentioned above, we introduced the MRF module and MFPN feature fusion network, which not only improve the utilization of feature information but also enhance the model’s ability to detect person keypoints. The results of the ablation experiments, as shown in Table 3, indicate increases of 1.6% and 4.1% in AP and AR, respectively. Moreover, in order to enhance the saliency of critical feature information, we introduced the CCAM attention module to assign weights to different positions in the feature space, further improving the model’s segmentation performance (+1.5%), as shown in Table 4. Compared to other real-time HPE methods, CCAM-Person achieves competitive performance on the MS COCO 2017 and CrowdPose datasets, as shown in Tables 1 and Table 2.

Although CCAM-Person demonstrates excellent performance on most metrics, it does not reach the optimal inference speed. We attribute this to the inclusion of the attention mechanism and the complex interaction of feature information, which often come with complex structures and larger parameter sizes, leading to decreased runtime efficiency. While our efficiency is not at the highest level, it remains capable of fulfilling the real-time demands of a majority of tasks.

## Conclusion

Our work proposes a framework called CCAM-Person for person detection and pose estimation. Comparative experiments on large-scale public datasets with other real-time human pose estimation methods demonstrate competitive results in terms of regression accuracy and inference speed. Through improvements in the network structure and information processing modes of the YOLOv8x-pose model, our method achieves breakthroughs in accuracy indicators. In future work, we will attempt more improvement designs based on these foundations to streamline the model structure and improve the model’s inference speed. This may include incorporating recent technologies such as ReXNet^7^ and PAGCP pruning^57^. Additionally, we also consider the application of the designed CCAM attention mechanism to other tasks such as image segmentation and object tracking.

## Data Availability

The datasets analyzed during the current study are available in the repositories of CrowdPose (https://github.com/Jeff-sjtu/CrowdPose.git) and MS COCO (https://cocodataset.org/).
